# Characterization of a leukocidin identified in *Staphylococcus pseudintermedius*

**DOI:** 10.1371/journal.pone.0204450

**Published:** 2018-09-27

**Authors:** Mohamed A. Abouelkhair, David A. Bemis, Richard J. Giannone, Linda A. Frank, Stephen A. Kania

**Affiliations:** 1 Department of Biomedical and Diagnostic Sciences, University of Tennessee College of Veterinary Medicine, Knoxville, Tennessee, United States of America; 2 Department of Bacteriology, Mycology and Immunology, Faculty of Veterinary Medicine, University of Sadat City, Menoufia, Egypt; 3 Chemical Sciences Division, Biological Mass Spectrometry, Oak Ridge National Laboratory, Oak Ridge, Tennessee, United States of America; 4 Department of Small Animal Clinical Sciences, College of Veterinary Medicine, University of Tennessee, Knoxville, TN, United States of America; Pusan National University, REPUBLIC OF KOREA

## Abstract

Bacterial infections from *Staphylococcus pseudintermedius* are the most common cause of skin infections (pyoderma) affecting dogs. Two component pore-forming leukocidins are a family of potent toxins secreted by staphylococci and consist of S (slow) and F (fast) components. They impair the innate immune system, the first line of defense against these pathogens. Seven different leukocidins have been characterized in *Staphylococcus aureus*, some of which are host and cell specific. Through genome sequencing and analysis of the *S*. *pseudintermedius* secretome using liquid chromatography mass spectrometry we identified two proteins, named “LukS-I” and “LukF-I”, encoded on a degenerate prophage contained in the genome of *S*. *pseudintermedius* isolates. Phylogenetic analysis of LukS-I components in comparison to the rest of the leukocidin family showed that LukS-I was most closely related to *S*. *intermedius* LukS-I, *S*. *aureus* LukE and LukP, whereas LukF-I was most similar to *S*. *intermedius* LukF-I *S*. *aureus* gamma hemolysin subunit B. The killing effect of recombinant *S*. *pseudintermedius* LukS-I and LukF-I on canine polymorphonuclear leukocytes was determined using a flow cytometry cell permeability assay. The cytotoxic effect occurred only when the two recombinant proteins were combined. Engineered mutant versions of the two-component pore-forming leukocidins, produced through amino acids substitutions at selected points, were not cytotoxic. Anti-Luk-I produced in dogs against attenuated proteins reduced the cytotoxic effect of native canine leukotoxin which highlights the importance of Luk-I as a promising component in a vaccine against canine *S*. *pseudintermedius* infections.

## Introduction

*S*. *pseudintermedius* is the primary cause of pyoderma (skin infection), the most common canine dermatologic disease, and is also associated with urinary tract infections, wound and surgical site infections, external ear otitis, abscess formation, mastitis and endocarditis [1–3]. Approximately 30–35% of the *S*. *pseudintermedius* isolates tested in our University of Tennessee College of Veterinary Medicine Bacteriology Laboratory from patients are methicillin-resistant (MRSP) and high levels of resistance occurs in other regions of the United States [3]. The vast majority of MRSP are multidrug resistant and there are increasing numbers of pandrug-resistant isolates [3–5].

Alternative approaches to control staphylococcal infections, such as vaccines, have been difficult to develop. This is likely rooted in the ability of the bacteria to neutralize and/or destroy important components of their host defenses. Some staphylococcal toxins impact the innate immune system, the first line of defense against this pathogen [6–8]. Antibody-mediated toxin neutralization may contribute to a strategy for immunotherapeutic prevention of current and recurrent infections [6, 9].

Leukocidins are a family of potent toxins contributing to the pathogenicity of staphylococci [10]. Leukocidins consist of two classes of proteins designated as S and F subunits [11–13] based on their chromatographic elution properties where S and F stand for slow and fast-eluting proteins, respectively [14, 15]. The subunits are produced and secreted separately. The S-component recognizes a receptor on the host cell, conferring high-affinity binding to the cell surface after which the F component is recruited to form octameric beta-barrel pores that penetrate the cell lipid bilayer into the plasma membrane leading to ion influx and efflux, apoptosis and ultimately cell death [11–13].

A total of seven different bicomponent pore-forming toxins (BCPFTs) have been identified and characterized in *Staphylococcus aureus* including HlgAB, LukMF, HlgCB, LukAB/HG, LukED, Panton-Valentine leukocidins (LukSF-PV/PVL), and LukPQ, some of which are host and cell specific [6]. The encoding genes are located chromosomally (*hlgAB* and *hlgCB* and *lukAB/HG)*, prophage associated (*pvl*, *lukPQ* and *lukMF)* or reside on a pathogenicity island (*LukED)* [6–8, 11–13].

A bi-component toxin (LukS-I + LukF-I) from *Staphylococcus intermedius* was identified and characterized previously [16]. Descloux et al [17] has reported the presence of a leukocidin encoding gene (LukS-I) in genomes of 15 different *S*. *pseudintermedius* strains including (*S*. *pseudintermedius* type strain CCUG49543T) isolated from dogs without characterization of the actual protein function.

In a previous study Karauzum et al. rationally designed mutants of *S*. *aureus* LukS-PV and LukF-PV subunits [18]. They tested mutant versions of LukS-PV with a series of amino acids substitutions and found that LukS-mut9, with T28F/K97A/S209A, was highly immunogenic and non-cytotoxic when mixed with LukF-PV[18]. Rabbit immunoglobulin raised against LukS-PV reduced the cytotoxic effect of canonical combinations (gamma hemolysin A and B subunits, gamma hemolysin C and B subunits and LukE and LukD), non-canonical pairs (gamma hemolysin B subunit and LukE, gamma hemolysin C subunit and LukD and gamma hemolysin A subunit and LukD) on polymorphonuclear leukocytes (PMNs) [19]. LukS-mut9 vaccines significantly protected in a mouse model of *S*. *aureus* USA300 sepsis and this effect could also be achieved by passive transfer of rabbit anti-LukS-mut9 antisera [18].

The purpose of the present study was to analyze the secretome of *S*. *pseudintermedius* by mass spectrometry (MS) to determine the abundance of secreted Luk-I, characterize the cytotoxic effect of recombinant *S*. *pseudintermedius* Luk-I on canine PMNs and develop attenuated, nontoxic LukS-I and LukF-I. Attenuated LukS-I and LukF-I were tested for their antigenicity and PMN killing. Furthermore, antibody raised against *S*. *pseudintermedius* attenuated Luk-I in clinically healthy dogs was evaluated for its ability to neutralize wild-type LukS-I and LukF-I. The results from this study suggest that *S*. *pseudintermedius* LukS-I and LukF-I may serve as key components in a vaccine or as part of an immunotherapeutic approach.

## Materials and methods

### Ethics statement

Experimental protocols were reviewed and approved by the University of Tennessee Institutional Animal Care and Use Committee (IACUC) including obtaining blood samples of dog (2474–0716) and injecting dogs with recombinant protein for producing antibodies (2572–1217).

### Bacterial strains, plasmids and growth conditions

The *S*. *pseudintermedius* strains used in this study, representing the most common multilocus sequence types (ST) previously reported in the United States [3, 4, 20], included 06–3228 (ST68), 08–1661 (ST71) and NA45 (ST84) [3, 4, 20]. Strains 06–3228 and 08–1661 were isolated at the University of Tennessee, College of Veterinary Medicine Bacteriology Laboratory. Strain NA45 was a gift of Faye Hartmann of the University of Wisconsin, School of Veterinary Medicine.

Plasmid construct pMA- attenuated *LukS-I*-M and pMA- attenuated *LukF-I*-M, each containing a mutated, synthetic *S*. *pseudintermedius* gene (designed as described below) with BamHI/NotI cloning sites, was obtained commercially (Life Technologies Corp., Carlsbad, CA).

Bacterial colonies grown on blood agar plates were inoculated into 5ml of sterile trypticase soy broth (TSB) (BD Biosciences, San Jose, CA Cat No. RS1-011-21) and incubated overnight at 37°C with shaking at 225 rpm (Excella E24 Incubator Shaker, New Brunswick Scientific). Fifty microliters of overnight culture were inoculated into 5ml of fresh, sterile TSB to initiate log-phase bacterial cultures. Bacteria were grown at 37°C with shaking at 225 rpm until an optical density of OD _600_ = 0.4–0.6 was reached.

### LC-MS/MS analysis of *S*. *pseudintermedius* supernatant

Log-phase bacterial cultures of 06–3228, 08–1661 and NA45 were centrifuged at 10,000 x g for 30 minutes at 4°C (Sorvall RC-5C Plus Super Speed Centrifuge) and the supernatant was collected and passed through a 0.45μm filter (Whatman, GE Healthcare Lifesciences, Pittsburgh, PA). The filtrate was concentrated using a centrifugal filter (EMD Millipore Corp., Billerica, MA) and stored at -20°C until further analysis. Samples interrogating *S*. *pseudintermedius* supernatant/ secretome were prepared for shotgun LC-MS/MS analysis as previously described [21].

Peptides were separated and analyzed with a 2-step MudPIT LC-MS/MS protocol (salt cuts of 50 and 500 mM ammonium acetate) over a 4-hr period then measured with a hybrid LTQ XL-Orbitrap (Thermo Scientific, Waltham, MA) mass spectrometer (MS) at Oak Ridge National Laboratories, Oak Ridge, Tennessee, USA[22]. Peptide fragmentation spectra were searched against sample-specific proteome databases (strains 06–3228, 08–1661 and NA45). Matching peptides (FDR < 1%) were assigned to proteins [23] and resulting secretomes compared. The percent coverage of detected proteins was calculated by dividing the number of amino acids in all found peptides by the total number of amino acids in the entire protein sequence.

### Bioinformatics analysis

Multiple sequence alignment (MSA) of mature LukS-I and LukF-I subunits of *S*. *pseudintermedius* 06–3228 strain with corresponding units in other leukotoxins (**Table 1)** was performed and a rooted phylogenetic tree (UPGMA (unweighted pair group method with arithmetic mean)) of Luk-I was generated with Geneious version 11.0.3 [24] with *S*. *pseudintermedius* protein A serving as an outgroup.

**Table 1:** Leuoktoxin subunits used in the rooted phylogenetic tree.

Protein name	Species	Accession Number	Amino acid Length
bi-component leucocidins LukPQ subunit Q	*Staphylococcus aureus*	WP_086037611.1	326
bi-component leucocidins LukPQ subunit P	*Staphylococcus aureus*	WP_086037612.1	311
bi-component leucocidins LukED subunit E	*Staphylococcus aureus*	WP_000473596.1	311
bi-component leucocidins LukED subunit D	*Staphylococcus aureus*	WP_099821693 .1	327
bi-component leucocidins LukMF subunit M	Staphylococcus aureus	WP_000476437.1	308
bi-component leucocidins LukMF subunit F	*Staphylococcus aureus*	WP_000694885 .1	322
bi-component leucocidins LukSF- PV subunit LukS-PV	*Staphylococcus aureus*	WP_000239544 .1	312
bi-component leucocidins LukSF- PV subunit LukF-PV	*Staphylococcus aureus*	WP_024937002 .1	327
bi-component leucocidins Hlg-AB subunit Hlg-A	*Staphylococcus aureus*	WP_000594519.1	309
bi-component leucocidins Hlg-AB subunit Hlg-B	*Staphylococcus aureus*	WP_000783426 .1	325
LukS-I	*Staphylococcus pseudintermedius*	WP_014613568.1	310
LukF-I	*Staphylococcus pseudintermedius*	WP_014613567 .1	326

In order to identify the unique residues in S-component of *S*. *pseudintermedius* Luk-I that may shape the protein function and specificity, a multiple alignment of amino acid sequences of the DR4 region in the rim domain (an important region for S-component receptor binding) of *S*. *aureus* LukE, HlgA, LukM and LukP, *S*. *pseudintermedius* and *S*. *intermedius* LukS-I was performed.

The bacterial localization prediction tool, PSORTb version 3.0.2 (http://www.psort.org/psortb/)[25] was used to determine the topology and domain structure of LukS-I and LukF-I.

*S*. *pseudintermedius* LukS-I and LukF-I modeling and binding site prediction was performed using Protein Homology/analogY Recognition Engine V 2.0 (Phyre^2^) (http://www.sbg.bio.ic.ac.uk/phyre2) [26] and the 3DLigandSite (http://www.sbg.bio.ic.ac.uk/3dligandsite/)[27] using *S*. *aureus* LukSF-PV, LukED and LukPQ as a basis to predict the critical amino acids for protein function. PHAST (http://phast.wishartlab.com/index.html) and PHASTER [28, 29] (http://phaster.ca/) were used for prophage detection in a total of 22 *S*. *pseudintermedius* isolates.

### Polymerase chain reaction (PCR) amplification of *LukS-I and LukF-I*

Bacteria from a single colony of *S*. *pseudintermedius* strain 06–3228 obtained from blood agar plates were grown in TSB at 37°C with 225 rpm shaking. DNA was extracted using a MO BIO DNA Isolation Kit (QIAGEN Inc. Cat No.12224-50) according to the manufacturer’s instructions. Oligonucleotide primers (Integrated DNA Technology, Coralville, USA) (**Table 2)** were designed using a PrimerQuest Tool (https://www.idtdna.com/Primerquest/Home/Index) based on the genomic sequence of *S*. *pseudintermedius* strain 06–3228 (20). The native *LukS-I* and *LukF-I* open reading frames (ORF) (933 and 981 bp, respectively) without the regions encoding the predicted N-terminal signal peptide were amplified from *S*. *pseudintermedius* 06–3228 genomic DNA and the ORF of mutant *LukS-I* and *LukF-I* were amplified from pMA-*attenuated LukS-I*-M and pMA-*attenuated LukF-I*-M plasmids (Life Technologies Corp., Carlsbad, CA), respectively (**Table 3)**. PCR was performed using taq polymerase (rTaq, Takara, Cat No. R004) and the following cycling conditions were performed: initial denaturation at 95°C for 90 seconds, 30 cycles of denaturation at 94°C for 30 seconds, annealing at 55°C for 30 seconds and extension at 72°C for 1 minute followed by a final extension at 72°C for 5 minutes. All ORFs were amplified without a histidine tag because pETBlue-2 allowed T7lac promoter-based expression of target genes with C-terminal histidine tagged sequences. PCR products were Sanger sequenced at The University of Tennessee Genomics Core facility.

**Table 2:** Primers used in this study to amplify recombinant wild type and attenuated *LukS-I and LukF-I* from *Staphylococcus pseudintermedius*.

***LukS-I*-forward**	GCATGA**GGATCC**GGTAAAAAATAAATTATTAGCCGCAACA
***LukS-I*-reverse**	GCATGA**GCGGCCGC**ATTATGCCCCTTTACTTTAATTTCGTG
***LukS-I*-M forward**	GCATGA**GGATCC**AAGGCCACGTGTCTTGTC
***LukS-I*-M reverse**	GCATGA**GCGGCCGC**CCCATGAGGCCAGTCTTG
***LukF-I*-forward**	GCATGA**CTCGAG**AAAAGAATGGCTAATCAAATTACACCTGTATCTG
***LukF-I*-reverse**	GCATGA**GGATCC**TTAGTGATGGTGATGGTGATGTACTGTATGCTGATCCCAATCAA
***LukF-I*-M forward**	GCATGA**GGATCC**GGATCCAATGAAAATAAGCAAAGTTATC
***LukF-I*-M reverse**	GCATGA**GCGGCCGC**GCGGCCGCTGATGGGTTTTTT

NotI, XhoI and BamHI restriction sites are underlined.

**Table 3:** Plasmids and competent cells used to clone and express recombinant wild type and attenuated *Staphylococcus pseudintermedius* LukS-I and LukF-I.

Plasmid/ Bacteria	Expressed Gene	Source
pMA-*LukS-I*-M	Contain attenuated *S*. *pseudintermedius LukS-I* (*LukS-I*-M)	Synthetic gene, Life Technologies Corp., Carlsbad, CA
pMA-*LukF-I*-M	Contain attenuated *S*. *pseudintermedius LukF-I* (*LukF-I*-M)	Synthetic gene, Life Technologies Corp., Carlsbad, CA
pETBlue-2	LukS-I and LukS-I-M and LukF-I-M expression with blue/white screening and C-terminal HSV and His tagged sequences	Novagen, Madison, WI
Dh5-alpha (DE3) pLacI	Cloning and recombinant LukS-I and LukS-I-M and LukF-I-M protein expression	Novagen, Madison, WI
pKLAC2	An integrative expression vector of *S*. *pseudintermedius* LukF-I in yeast	New England Biolabs, Ipswich, MA
*Kluyveromyces* lactis	An expression host of *S*. *pseudintermedius* LukF-I	New England Biolabs, Ipswich, MA

### Cloning, expression and purification of recombinant native and attenuated *Luk-I*

To clone *S*. *pseudintermedius* native and mutant LukS-I and mutant LukF-I, their PCR products were digested with *NotI* and *BamHI*, then ligated into pETBlue-2 (Novagen, Cat No .70674) and transformed into DH5-alpha *E*. *coli* chemically-competent cells (**Table 3)** (New England BioLabs Inc., Cat No .C2987I) by heat shock. The DH5-alpha *E*. *coli* were plated on LB agar plates with 100 μg/mL ampicillin. The plasmid constructs were transformed into DE3 pLacI *E*. *coli* chemically-competent cells (**Table 3)** (Novagen, Cat No .70623) by heat shock and the pLacI *E*. *coli* were plated on LB agar containing 50μg/ml ampicillin and 20μg/ml chloramphenicol.

To express recombinant *S*. *pseudintermedius* native and mutant LukS-I and mutant LukF-I, a single colony of DE3 pLacI *E*. *coli* was inoculated into LB broth containing 50μg/ml ampicillin and 20μg/ml chloramphenicol and bacteria grown overnight at 37°C with 225 rpm shaking. LB broth containing 50μg/ml ampicillin and 20μg/ml chloramphenicol was inoculated with a 1:100 dilution of overnight culture and grown at 37°C with 225 rpm shaking until a 600 nm optical density of between 0.4 and 0.6 was reached. Protein expression was induced by addition of 1 mM Isopropyl β-D-1-thiogalatopyranoside (IPTG) (Teknova, Cat. No. I3431) and bacteria were grown for 4 hr at 30°C with shaking at 225 rpm. Bacterial cultures were centrifuged at 12,000 x g for 5 min in 5 ml of protein extraction reagent (BugBuster, Novagen Cat No. 70584) and 20 μl of 100X protease inhibitor (Cocktail Set III, EDTA-Free Calbiochem, Cat No. 539134) and incubated for 30 min at 37°C in a shaking incubator at 225 rpm. Bacteria were pelleted by centrifugation at 12,000 x g for 45 min at 4°C. Recombinant proteins were purified using affinity purification (His Ni-NTA Spin Purification Kit, Thermo Scientific, Cat No. 88228).

Recombinant native LukF-I was cloned using an integrative expression vector (pKLAC2) and expressed in *Kluyveromyces lactis* (New England Biolabs, Cat No. E1000S). Recombinant protein was purified from *K*. *lactis* supernatant using affinity purification (His Ni-NTA Spin Purification Kit, Thermo Scientific, Cat No. 88228). Protein concentrations were determined using a bicinchoninic acid (BCA) assay (Thermo Scientific, Cat No.23227).

### SDS-PAGE and western blot

Protein samples were resolved by SDS-PAGE in 4–12% polyacrylamide gels (Invitrogen, Cat No. NP0322BOX) and electrophoretically transferred onto nitrocellulose membranes (Thermo Scientific, Cat No. 77010). The blots were blocked overnight in 5% (wt/vol) nonfat dried milk powder in 0.05% polyethylene glycol sorbitan monolaurate (Tween 20) containing phosphate buffered saline (PBS-T) at 4°C. The blocked membranes were incubated with a 1:2,000 dilution of horseradish peroxidase (HRP)-conjugated anti-6xhis tag monoclonal antibody (Thermo Scientific, Cat No. MA1-21315-HRP) in 0.05% PBS-T for 1 h with 225 rpm shaking at room temperature. After five washes with 0.05% PBS-T bound antibodies were detected using chloronaphthol substrate solution (Thermo Scientific, Cat No. 34012).

### Preparation of canine anti- *S*. *pseudintermedius* Luk-I

Recombinant attenuated LukS-I and LukF-I produced in *E*.*coli* were purified using affinity chromatography (as above) and endotoxin concentrations were measured using a chromogenic LAL Endotoxin Assay Kit (Genscript, Cat. No. L00350). Recombinant attenuated LukS-I and LukF-I at 20 μg each / 0.5 cc in phosphate buffered saline (PBS) (pH 7.2) were injected in the lateral thorax by the subcutaneous route, into three clinically normal dogs. Injections were given once every 7 days for a total of three injections with a control dog receiving PBS (pH 7.2) only. Blood (6 cc) was collected from a jugular vein 4 times, on days -7, 8, 15 and 29. The collected blood was left undisturbed at room temperature for 30 min followed by centrifugation at 2,000 x g for 10 min in a refrigerated centrifuge.

### Enzyme-linked immunosorbent assay

For measurement of recombinant protein antigenicity, attenuated LukS-I and LukF-I were coated separately onto ELISA plates (Corning, Cat No. 3590) at 2μg/ml in PBS (pH 7.2). The plates were washed with 0.05% PBS-T and incubated with two-fold serial diluted serum from dogs (injected with recombinant proteins) for 1 h at 37°C, then bound IgG was detected using HRP-conjugated goat anti-dog IgG heavy and light chain (Bethyl Laboratories, Inc. Cat No. A40-123-1). ELISA assays plates were washed three times with PBS-T between all incubations, bound antibodies were detected using TMB substrate (Thermo Scientific, Cat No. N301), reactions were stopped with 0.18 M sulphuric acid and optical density read at 450 nm on a plate reader (Bio TEK, EL800). The experiment was repeated a minimum of three times and a p-value of <0.05 was considered significant for all the experiments unless otherwise stated.

### PMN cell permeability assay

Canine blood was collected from healthy dogs using a sterile blood collection system with EDTA anti-coagulant (BD Vacutainer). Then, 600 μl of dog blood was added to 1 ml of red blood cell lysing buffer (Sigma-Aldrich, Cat No. R7757-100ML) for 30 min at 37°C in 15 ml sterile plastic tube, centrifuged and re-suspended in 1ml RPMI medium supplemented with 10% fetal bovine serum. PMNs were incubated with recombinant proteins (LukS-I and LukF-I, LukS-I alone, LukF-I alone, attenuated LukS-I, attenuated LukF-I and 1:2 S. *pseudintermedius* 06–3228 supernatant) in a volume of 500 μl in RPMI medium supplemented with 10% fetal bovine serum in a 5% CO_2_ incubator for 30 min. Supernatant of *S*. *pseudintermedius* 06–3228 was harvested at log phase to test the toxic effect of secreted LukS-IR. PMNs were stained with 1 μl of Sytox Green (Life technologies, Inc. Cat No. 1776406) for 30 min, washed with PBS (pH 7.2) twice and analyzed using a flow cytometer (Attune acoustic focusing cytometer). In order to measure the protective effect of anti-LukS-IR on canine PMNs, recombinant LukS-I and LukF-I were incubated with canine anti-attenuated LukS-I LukF-I at a dilution of 1:100 for 30 min at 37°C, then tested with the cell permeability assay as previously described.

### Biotin labeling of *S*. *pseudintermedius* wild type and attenuated Luk-I and single components

Purified recombinant *S*. *pseudintermedius* wild type and attenuated LukS-I and LukF-I at 500 μg/ml in PBS (pH 7.2) were incubated with 50μl of 10mM EZ-Link Sulfo-NHS-LC-Biotin reagent (equal to 20 fold molar excess of biotin) (Thermo Scientific, Cat No. 21327) for 30 min at room temperature. Excess biotin was removed using an Amicon Ultra-0.5 Centrifugal Filter Unit with a 30 kDa molecular weight cut-off (Milipore sigma, Cat No. UFC5030). The biotin-labeled proteins were stored at -20°C until further use.

To test the binding of wild type and attenuated LukS-I and LukF-I to canine PMNs, biotin labelled recombinant proteins were incubated with PMNs from a clinical healthy dog for 30 minutes at room temperature, the cells were washed, then PMNs were incubated with 1:500 dilution of avidin-FITC conjugate (Sigma-Aldrich, A2050) at room temperature for 30 minutes in the dark. Unbound conjugate was removed by washing and the amount of binding was determined using a flow cytometer (Attune acoustic focusing cytometer).

### Statistical analysis

Data was analyzed using ANOVA tests and followed by post hoc multiple comparisons with Tukey’s adjust. Diagnostic analysis was performed on residuals for checking normality and equal variance assumptions. Rank data transformation was applied when assumptions were violated. Statistical significance was identified at the level of 0.05. Analyses were conducted in JMP pro 14 for Windows (SAS institute Inc., Cary, NC).

## Results

### LC MS/MS data analysis of *S*. *pseudintermedius* culture supernatant

Secreted proteins, were isolated and digested to peptides with trypsin and analyzed by LC-MS/MS. The high mass accuracy of intact peptides and their fragmentation profiles were searched against genome-derived amino acid sequences for each protein in the sequenced genome of each strain. Identified peptides were then mapped back to their respective proteins and proteins quantified by both sequence coverage as well as area-under-the-curve abundance. Using this semi-quantitative data, along with categorizing each protein by their predetermined cellular location (pSORT), we were able to identify both LukS-I and LukF-I proteins in supernatant at relatively high levels, i.e. rank 5 (LukF-I) and rank 7 (LukS-I) out of 92 total extracellular proteins identified (PSORT: extracellular, membrane/cell wall-associated, location unknown), putting both in the top 10% of the extracellular dataset. These MS identification confirmed the extracellular existence of these proteins in all virulent strains and that they are indeed expressed at relatively high-levels, thus serving as candidates for the further analysis (presented here and on-going).

The percent coverage of LukS-I and LukF-I were calculated by dividing the number of amino acids in all found peptides by the total number of amino acids in the mature protein sequence **Table 4.**

**Table 4:** LC MS/MS analysis of *S*. *pseudintermedius* culture supernatant.

Strain/Protein	Coverage
**Strain 06–3228**	
LukS-I	10.0
LukF-I	39.3
**Strain 08–1661**	
LukS-I	56.2
LukF-I	46.0
**Strain NA45**	
LukS-I	59.4
LukF-I	62.3

LukS-I and LukF-I were secreted by *S*. *pseudintermedius* 06–3228, 08–1661 and NA45. Secretome proteins were compared among the three isolates using their respective genomes as reference databases, the percent coverage were calculated by dividing the number of amino acids in all found peptides by the total number of amino acids in the mature protein sequence.

### Characterization of *S*. *pseudintermedius* Luk-I

Multiple sequence alignment (MSA) analysis showed that Luk-I is conserved among *S*. *pseudintermedius* strains including 06–3221, 08–1661 and NA45 with amino acid identities over 99.4% between strains.

A 14.9 kb incomplete prophage (similar to Φ Staphy_96_NC_007057) was identified in the genome of *S*. *pseudintermedius* 06–3228. A BLAST search of Φ Staphy_96_NC_007057 using complete genome sequences of *S*. *pseudintermedius* strains available in the GenBank database and others sequenced in our lab but not yet published, revealed that approximately 7 Kb of the phage are present in all of *S*. *pseudintermedius* isolates examined (a total of 22 isolates). They also contain the coding DNA sequences (CDS) of ascorbate-specific PTS system EII A, B and C components, probable L-ascorbate-6-phosphate lactonase UlaG (L-ascorbate utilization protein G), phosphoglycerate mutase family 2 and hypothetical protein.

**Fig 1:**
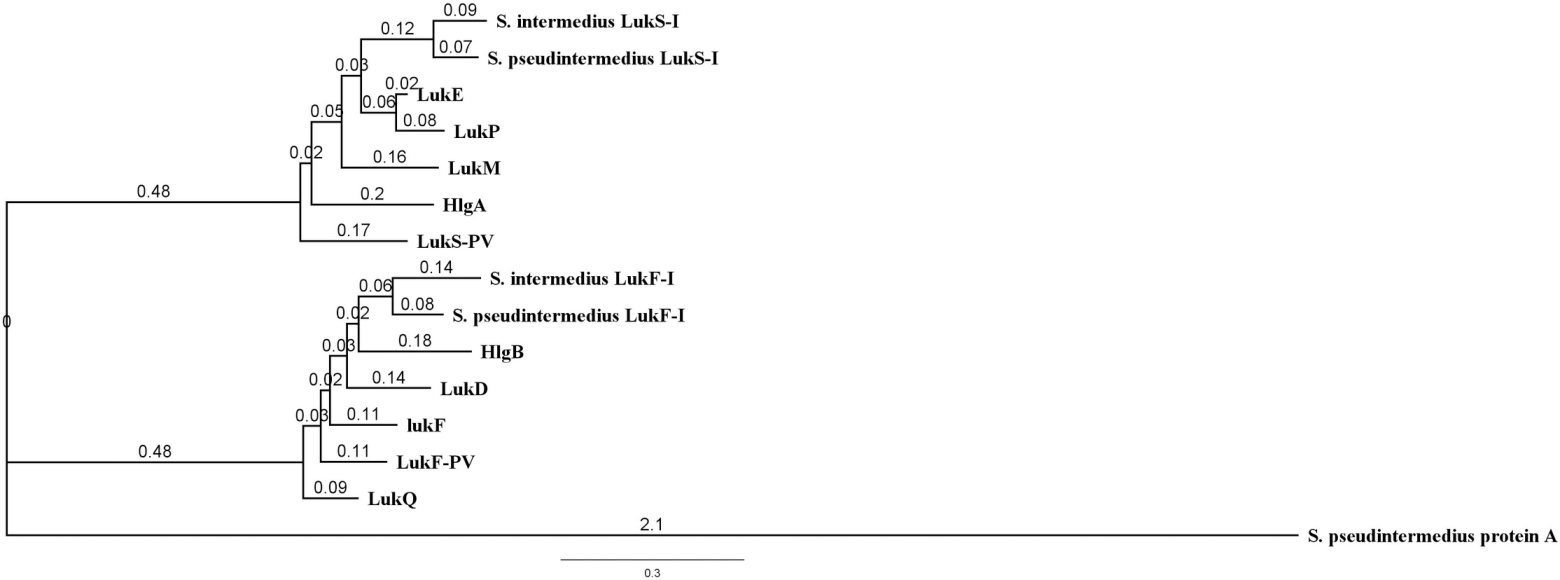
Phylogenetic tree based on amino acid sequences of mature leucocidins produced by *S*. *aureus*, *S*. *intermedius* and *S*. *pseudintermedius*. *S*. *pseudintermedius* LukS-I is closely related to *S*. *intermedius* LukS-I, *S*. *aureus* LukE and LukP. *S*. *pseudintermedius* LukF-I is closely related to *S*. *intermedius* LukF-I and *S*. *aureus* gamma hemolysin subunit B. *S*. *pseudintermedius* protein A was used as an outgroup.

Phylogenetic analysis of Luk-I in comparison to the entire leukocidin family showed that *S*. *pseudintermedius* LukS-I is closely related to *S*. *intermedius* LukS-I, *S*. *aureus* LukE and LukP. However, *S*. *pseudintermedius* LukF-I is closely related to *S*. *intermedius* LukF-I and *S*. *aureus* gamma hemolysin subunit B (Fig 1).

Multiple sequence alignment (MSA) analysis showed that Luk-I in *S*. *pseudintermedius* is a unique leukotoxin and that each functional component shares considerable similarity with other staphylococcal leukotoxin fractions (**Table 5)**. The protein sequence contains an N-terminal signal peptide sequence from positions 1 through 29 for LukS-I and 1 through 26 for LukF-I detected by SignalP 4.1 server [30].

**Table 5:** MSA of LukS-I and LukF-I subunits of *S*. *pseudintermedius* 06–3228 strain with corresponding units in other leukotoxins.

	**LukP**	**hlgA**	**LukE**	**LukM**	**LukS-PV**	***S*. *intermedius* LukS-I**
***S*. *pseudintermedius* LukS-I**	75.64%	73.08%	73.72%	68.49%	64.76%	85.48%
** **	**LukF-PV**	**LukQ**	**LukF**	**LukD**	**hlgB**	***S*. *intermedius* LukF-I**
***S*. *pseudintermedius* LukF-I**	73.60%	74.54%	73.46%	76.07%	72.62%	79.75%

**Fig 2:**
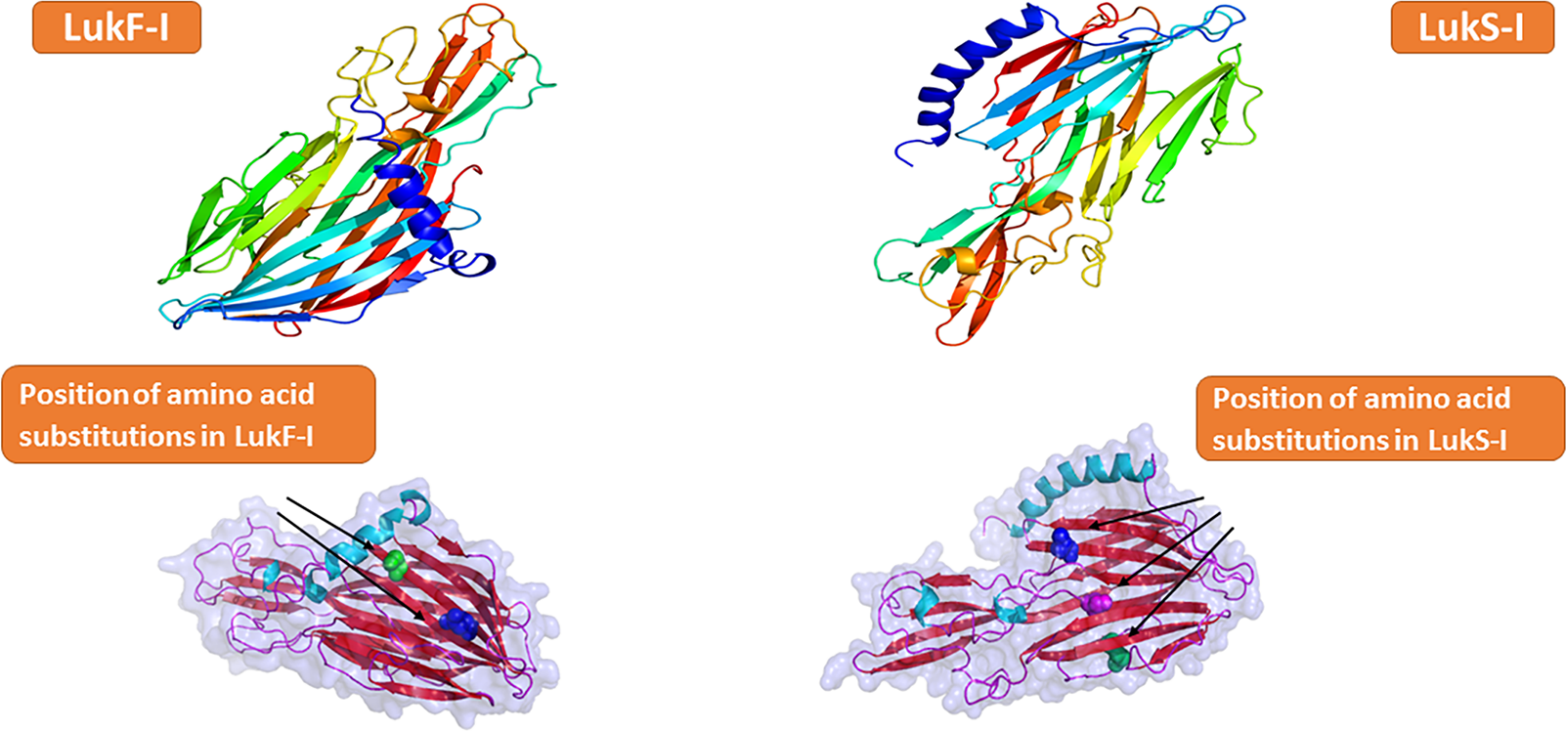
Ribbon representation of Wild-type and attenuated *S*. *pseudintermedius* LukS-I and LukF-I proteins. **a,** Color-ramped from the N terminus (blue) to the C terminus (red). The output structure was generated with Pymol.

**Fig 3:**
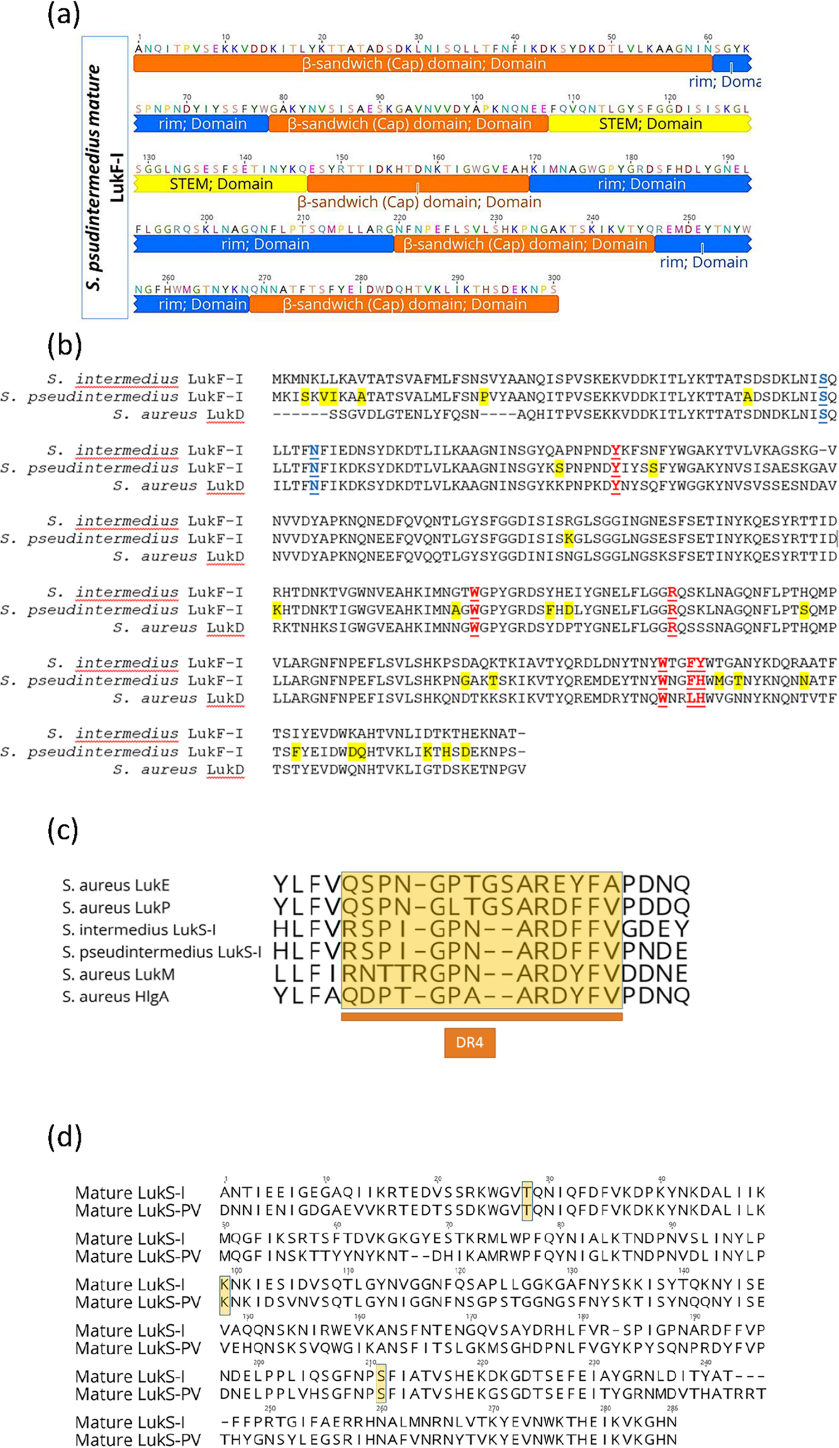
Unique residues in S and F-components of *S*. *pseudintermedius* Luk-I may shape the protein function and specificity. **A,** domain structure of *S*. *pseudintermedius* LukF-I consist of β-sandwich (cap) domain highlighted in orange color (1–60, 79–107, 147–169, 220–246 and 569–300), stem highlighted in yellow (108–146) and rim highlighted in blue (61–78, 170–219 and 247–268). **B,** Multiple sequence alignment (MSA) of *S*. *intermedius* and *S*. *pseudintermedius* LukF-I and *S*. *aureus* LukD. Geneious version 11.0.3 was used to generate the alignment. Residues unique to *S*. *pseudintermedius* LukF-I are highlighted yellow, residues identified as important for phospholipid interaction are shown in red and underlined text and residues important for oligomerization is highlighted in blue. **C,** MSA of amino acid sequences of the DR4 region (highlighted in yellow) in the rim domain (an important region for S-component receptor binding) of *S*. *aureus* LukE, HlgA, LukM and LukP, *S*. *pseudintermedius* LukS-I and *S*. *intermedius* LukS-I. The DR4 regions of *S*. *aureus* LukP and LukS-I of *S*. *pseudintermedius* and *S*.*intermedius* are almost identical, whilst that of LukM, HlgA and LukE are considerably different. **D,** MSA of amino acid sequences of *S*. *pseudintermedius* LukS-I and *S*. *aureus* LukS-PV showing the critical residues T28, K99 and S211 (highlighted in yellow) that interact with the corresponding F-component.

LukF-I model developed with the Phyre^2^ web portal, shows that LukF-I consist of β-strands organized as four antiparallel β-sheets and three very short α-helices (Fig 2). LukF-I, similar to the fold organization of other bicomponent pore-forming toxins, is arranged in three typical domains (β-sandwich (cap) domain (1–60, 79–107, 147–169, 220–246 and 569–300), stem (108–146) and rim (61–78, 170–219 and 247–268)) (Fig 3A). The rim domain has critical residues for membrane binding, the β-sandwich (cap) domain play a role in side by side residues interaction and oligomerization with S component, and the pre-stem domain is important for pores formation but not membrane-binding.

In *S*. *pseudintermedius* LukF-I, we identified Tyrosine (Y) 71, Tryptophan (W) 176, Arginine (R) 179, W256, Phenylalanine (F) 259 and histidine (H) 260 as a critical residues located in the rim domain and identified as important for phospholipid binding on the membranes of canine PMNs while Serine (S) 33 and Asparagine (N) 39 located in the β-sandwich (cap) domain as essential amino acids to interact with side residues of LukS-I which stimulate oligomerization. These residues are conserved among *S*. *intermedius* LukF-I and *S*. *aureus* LukD (Fig 3B).

MSA of amino acid sequences of the divergent region 4 (DR4) region in the rim domain (an important region for S-component receptor binding) of *S*. *aureus* LukE, HlgA, LukM and LukP, *S*. *pseudintermedius* LukS-I and *S*. *intermedius* LukS-I revealed that the DR4 regions of *S*. *aureus* LukP and LukS-I of *S*. *pseudintermedius* and *S*.*intermedius* are almost identical, whilst that of LukM, HlgA and LukE are considerably different (Fig 3C).

### Design of LukS-I and LukF-I with amino acid substitutions

We designed attenuated LukS-I and LukF-I with mutations away from the rim region because we need to disrupt the ability of LukS-I and LukF-I to interact side by side and form oligomers, a function critically required for cytolysis.

*S*. *pseudintermedius* LukS-I and *S*. *aureus* LukS-PV share the critical residues T28, K99 and S211 for interaction and oligomerization with the corresponding F-component [18], Guided by LuKS-I protein model developed by Phyre^2^, and MSA of amino acid sequences of *S*. *pseudintermedius* LukS-I and *S*. *aureus* LukS-PV (Fig 3D), an attenuated *S*. *pseudintermedius* LuS-I was designed with the following substitutions: T28F, K99A and S211A.

Moreover, we identified Serine (S) 59 and Asparagine (N) 65 in the mature LukF-I protein as essential residues for side-by-side interactions with LukS-I to form oligomers. To test that we designed attenuated LukF-I with the following substitutions S59D and N65A.

### Cloning, expression and purification of recombinant *S*. *pseudintermedius* LukS-I and LukF-I

**Fig 4:**
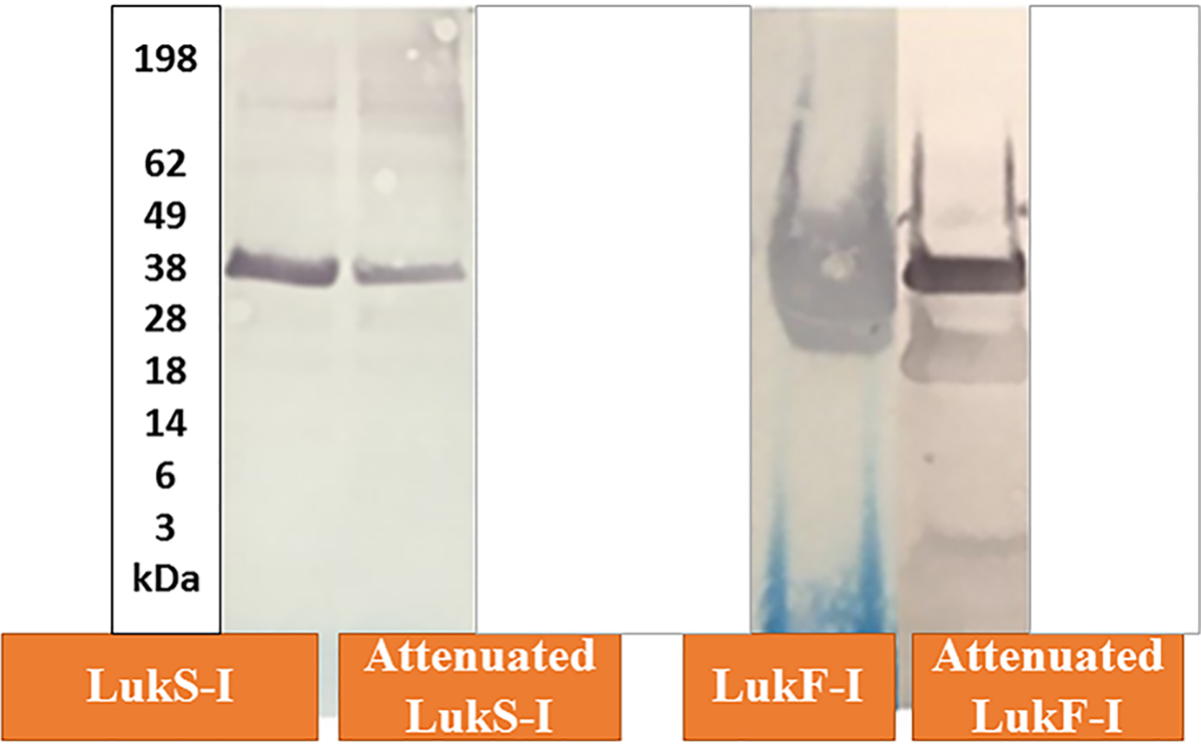
Western blot of recombinant *S*. *pseudintermedius* wild-type and attenuated LukS-I and LukF-I with HRP-conjugated anti-6xhis tag monoclonal antibody. The Molecular weights of LukS-I, LukF-I, attenuated LukS-I and attenuated LukF-I determined in western blots using pre-stained Protein Standard (x 1,000) were of the expected sizes 39.43, 39.12, 37.27 and 37.59 kDa, respectively.

Recombinant polyhistidine tagged wild type and mutant LukS-I and LukF-I were generated in *E*.*coli* and LukF-I was secreted in the culture supernatant of *K*. *lactis* using an integrative expression vector (pKLAC2). Recombinant proteins were purified using HisPur Ni-NTA resin under native conditions and eluted using an imidazole gradient. The molecular weights of LukS-I, LukS-I-M, LukF-I and LukF-I-M determined in western blots were of the expected sizes (39.43, 39.12, 37.27 and 37.59 kDa, respectively) (Fig 4). The endotoxin levels of purified recombinant, attenuated LukS-I and LukF-I were below 0.5 endotoxin units /mg protein.

### Attenuated Luk-I induces specific antibody responses

**Fig 5:**
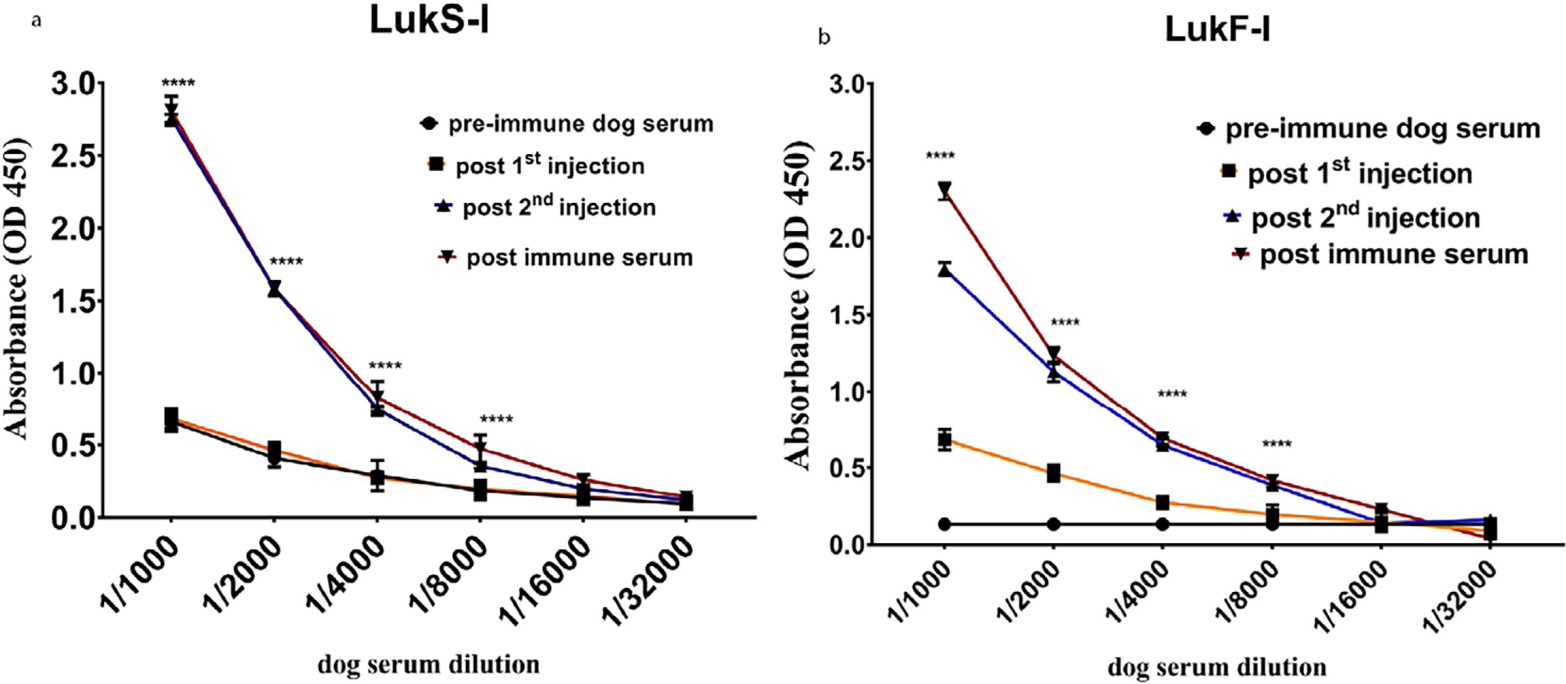
Canine antibody against attenuated LukS-I and LukF-I react with recombinant wild-type LukS-I and LukF-I. Antibodies against *S*. *pseudintermedius* wild-type LukS-I and LukF-I were detected using an indirect ELISA. Recombinant *S*. *pseudintermedius* LukS-I and LukF-I proteins were coated on ELISA plates, then incubated with two-fold serially diluted serum from dog vaccinated with the same proteins. High reactivity with LukS-I and LukF-I was seen from sera collected two weeks after 3^rd^ injetions of attenuated LukS-I (P <0.0001****) and LukF-I (P <0.0001****) compared to pre-injection sera. The values represent averages from three independent experiments.

Specific antibodies against *S*. *pseudintermedius* wild type and attenuated LukS-I and LukF-I were detected using an indirect ELISA, with sera collected on days -7, 8, 15 and 29, after the second injection of attenuated Luk-I (on day 15) and was higher on day 29 (P < 0.0001) compared to pre-injection control sera (Fig 5, S1 and S2 Tables).

### Luk-I kills canine PMNs

**Fig 6:**
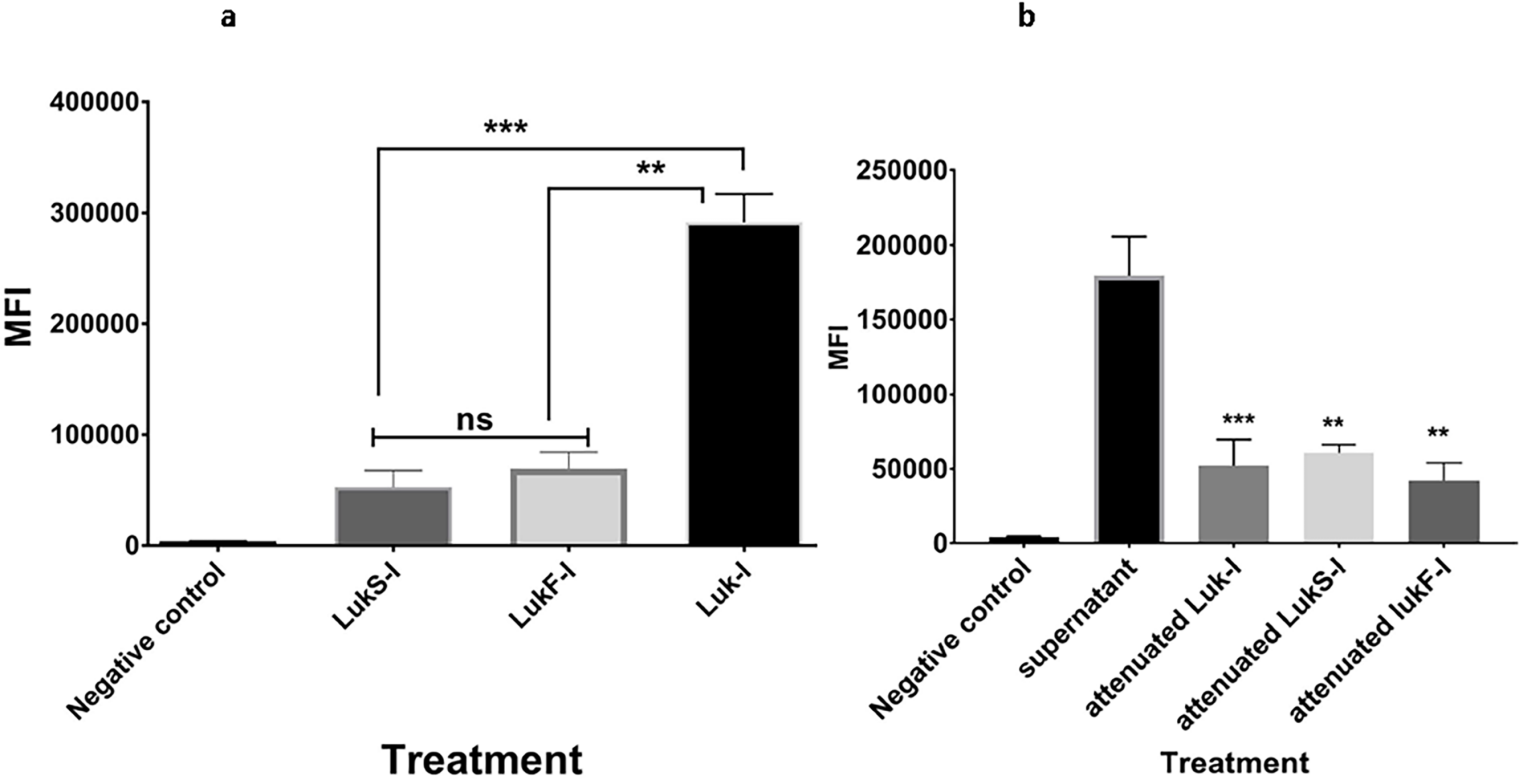
Cytotoxic effect of *S*. *pseudintermedius* recombinant Luk-I on canine PMNs. PMN permeability assay using Sytox Green to detect the cytotoxic effect of wild-type Luk-I on canine PMNs. (A) Luk-I significantly induced PMN killing after 30 min compared to that with wild-type LukS-I (P = 0.0330***) and LukF-I (P = 0.0468**). (B) A1:2 S. *pseudintermedius* 06–3228 supernatant dilution significantly induced PMN killing after 30 min compared to that with and attenuated LukS-I (P = 0.0276**), attenuated LukF-I (P = 0.0268**) and attenuated Luk-I treatments (P = 0.0044***). The mean fluorescent intensity (MFI) of all treatment were calculated based on average values from three independent experiments. (*P < 0.05 was considered significant). ns–Not significant.

Canine PMNs were highly susceptible to Luk-I with lysis induced within 30 minutes at a concentration of 200 ng of each leukototxin component (P <0.0001) and a 1:2 *S*. *pseudintermedius* 06–3228 supernatant dilution (P <0.0001) (Fig 6A, S3 and S4 Tables). Attenuated Luk-I or wild-type proteins alone showed a diminished killing effect on PMNs of dogs (Fig 6B).

### Amino acid substitutions in *S*. *pseudintermedius* LukS-I and LukF-I did not abolish leukotoxin binding to canine PMNs

**Fig 7:**
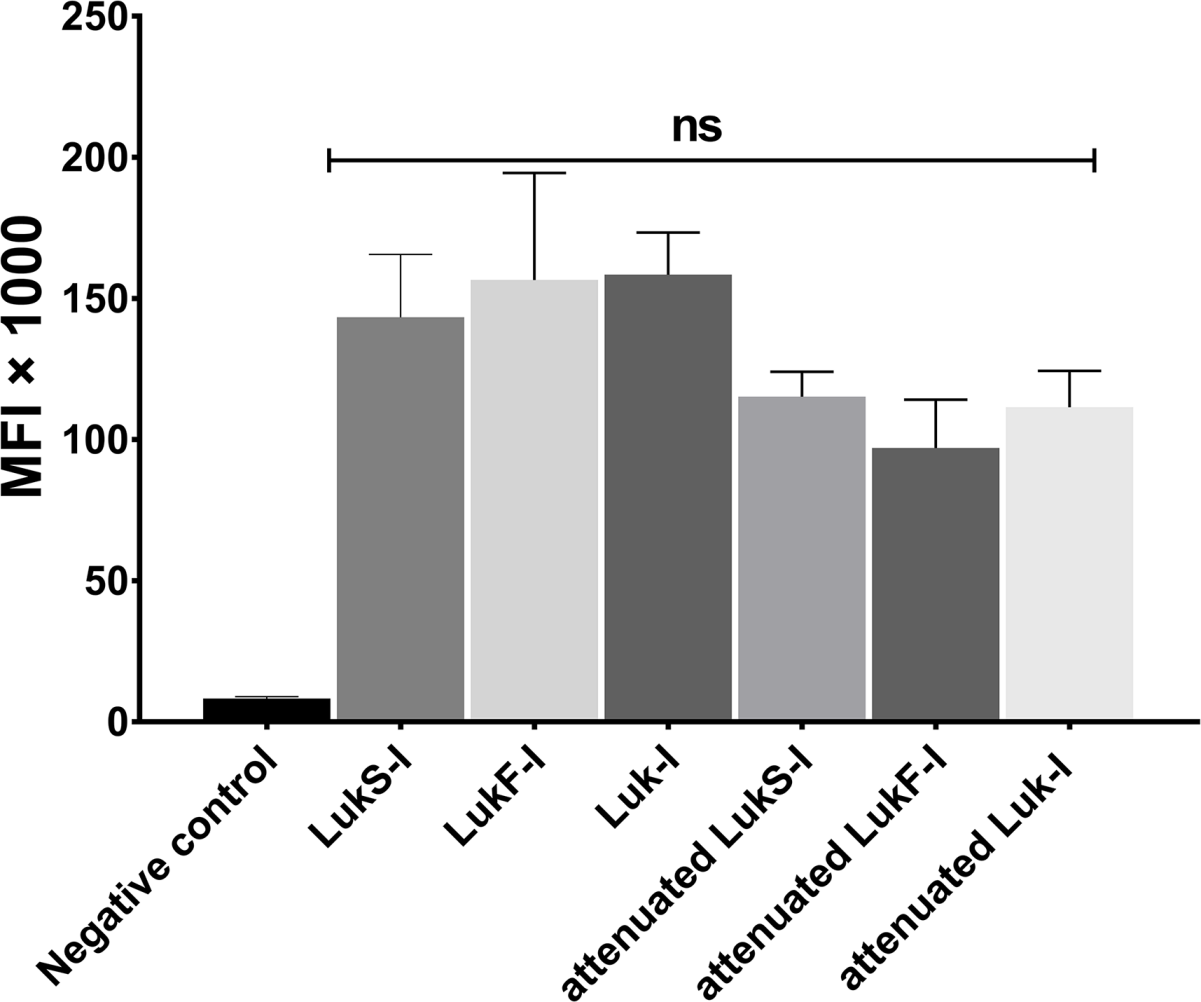
*S*. *pseudintermedius* wild-type and attenuated Luk-I binding to canine PMNs. Biotin labelled recombinant wild type and attenuated LukS-I and LukF-I were incubated with canine PMNs and their binding was detected using FITC conjugated avidin. MFI of the blank, wild-type and attenuated proteins were calculated based on average values from three independent experiments. All of the recombinant proteins bind to canine PMNs with no significant difference (ns).

To test the effect of critical amino acid substitutions on attenuated LukS-I and LukF-I binding to canine PMNs, biotin labelled recombinant wild type and attenuated LukS-I and LukF-I were incubated with canine PMNs and their binding detected using FITC conjugated avidin. No significant difference in MFI of attenuated or wild type recombinant LukS-I and LukF-I was found (P >0.05) (Fig 7 and S5 Table).

### Dog Anti-Luk-I reduced the cytotoxic effect of canine leukotoxin on PMNs

**Fig 8:**
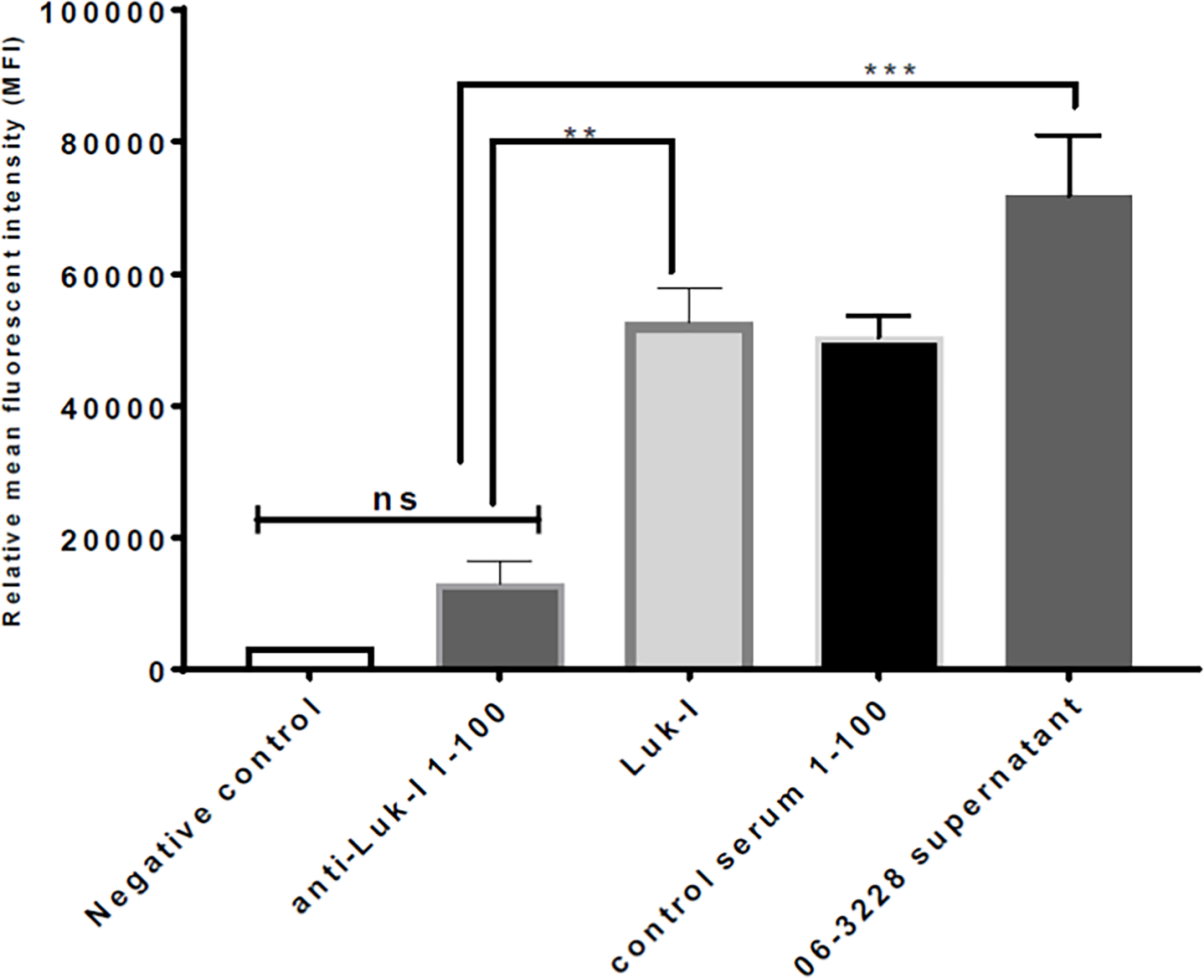
Dog antibody raised against attenuated LukS-I and LukF-I protects canine PMNs from the cytotoxic effect of Luk-I. The PMN permeability assay was performed. Luk-I preincubated with dog anti-Luk-I resulted in a significant reduction in the mean fluorescent intensity (MFI) compared with that of Luk-I treatment alone (P = 0.0036**) and with that of 06–3228 supernatant treatment alone (P = 0.0002***).

Dog anti-Luk-I at a dilution of 1:100 preincubated with Luk-I showed a significant reduction in mean fluorescent intensity (MFI) compared with Luk-I treatment alone (Fig 8A and 8B, S6 Table).

## Discussion

We identified a bicomponent leukotoxin, Luk-I, composed of LukS-I and LukF-I proteins that secreted by *S*. *pseudintermedius* and kill canine PMNs by pores formation and cell lysis. Luk-I, a member of the leukocidin family, is highly conserved among *S*. *pseudintermedius* isolates. Luk-I is associated with an incomplete prophage that occurs in degenerate form across all *S*. *pseudintermedius* isolates for which genomic sequence is available.

In accordance with its host distribution in a canine opportunistic pathogen, LukS-IR is cytotoxic against dog PMNs. This highlights the immune-evasive attribute of *S*. *pseudintermedius* Luk-I in the dog, in line with the assumed function of other phage-encoded leukocidins that similarly have a host-specific function and distribution. For example, LukMF is highly toxic to ruminant neutrophils and LukPQ preferentially kills horse neutrophils [6, 7, 31].

Based on the crystal structure of β-sandwich (cap) domain of *S*. *aureus* γ-hemolysin LukF component and *S*. *pseudintermedius* LukS-I and LukF-I model, we designed attenuated LukS-I and LukF-I with mutations away from the rim region. The attenuated LukS-I and LukF-I bind to canine leukocytes without causing any significant killing which indicates that mutations disrupt the ability of attenuated LukS-I and LukF-I to oligomerize, an essential function required for cytolysis. This observation is in agreement with the findings of Karauzum et al [18] with LukS-Mut9.

The low concentration of endotoxin in the recombinant proteins and the low toxicity exhibited by the attenuated protein produced in *E*. *coli* suggests that endotoxin did not play a role in neutrophil killing.

Expression of LukF-I in *E*.*coli* was not successful and for unknown reasons this protein appeared to be lethal to these bacteria; therefore, a yeast protein expression system *(K*. *lactis)* was used for this protein. *K*. *lactis* is considered the best alternative to bacterial expression systems. It efficiently produces recombinant proteins in culture supernatant that are also secreted by their native host [32–34]. *K*. *lactis* protein expression is driven by a variant of the strong PLAC4 promoter (PLAC2) [35] that lacks background expression in *E*.*coli* [32].

The high titer of Luk-I-specific, neutralizing antibodies produced in a clinically healthy dog injected with with attenuated LukS-I and LukF-I is similar to the antibody production that Karauzum et al [18] and Adhikari et al [19] observed with LukS-PV mutant (PVL- S subunit) named “LukS-mut9” injected in mice.

With widespread antimicrobial resistance, it is critical to find alternative approaches, such as vaccines, to control staphylococcal infections. Previous studies [36–38] concluded that a multivalent vaccine would likely work best in preventing infections caused by *S*. *aureus*. Taking the same factors into account, a potent and effective vaccine against *S*. *pseudintermedius* would involve identifying antigenic targets conserved among a wide variety of strains and sequence types. Understanding the nature of extracellular proteins and their role in virulence and pathogenesis is critical for vaccine development against *S*. *pseudintermedius* infection.

Using mass spectrometry and genomic information, it was possible to identify a unique leucocidin present in *S*. *pseudintermedius* isolates 06–3221, 08–1661 and NA45, representing the three clonal complexes that predominate in the United States [4].

## Conclusions

In conclusion, we describe a unique pore-forming toxin from *S*. *pseudintermedius*. To our knowledge this is the only leucocidin from a species other than *S*. *aureus* characterized to date. Mutant versions of the proteins had a reduced cytotoxic effect on dog PMNs and anti-Luk-I produced in dogs against attenuated proteins reduced the cytotoxic effect of wild type canine leukotoxin. Therefore, these mutants may serve as important components of a multivalent vaccine for prophylaxis or control of *S*. *pseudintermedius* infections. Such a vaccine may neutralize extracellular toxins responsible for host tissue destruction and immunosuppression and may help the host immune system control infections.

## Supporting information

S1 Table Canine antibody reactive with LukF-I, ELISA. (XLSX)

S2 Table Canine antibody reactive with LukS-I, ELISA. (XLSX)

S3 Table PMN cell permeability assay flow cytometry MFI. (XLSX)

S4 Table PMN cell permeability assay flow cytometry supernatant MFI. (XLSX)

S5 Table Leukotoxin binding to canine PMN flow cytometry. (XLSX)

S6 Table Dog Anti-Luk-I inhibition of canine leukotoxin cytotoxicity on PMNs. (XLSX)
